# Nutritional Immunity in Wound Infection: Unveiling the Role of Dietary Elements in Host–Pathogen Interaction

**DOI:** 10.1002/fsn3.70854

**Published:** 2025-09-02

**Authors:** Chaoming Chen, Xuanfan Hu, Da He, Xuemei He, Lan Shen

**Affiliations:** ^1^ Critical Care Center Southern Central Hospital of Yunnan Province (The First People's Hospital of Honghe State) Gejiu Yunnan China; ^2^ Ultrasonography Lab Southern Central Hospital of Yunnan Province (The First People's Hospital of Honghe State) Gejiu Yunnan China; ^3^ Infectious Disease Southern Central Hospital of Yunnan Province (The First People's Hospital of Honghe State) Gejiu Yunnan China; ^4^ Nephrology Department Southern Central Hospital of Yunnan Province (The First People's Hospital of Honghe State) Gejiu Yunnan China; ^5^ Critical Care Center Southern Central Hospital of Yunnan Province (The First People's Hospital of Honghe State) Gejiu Yunnan China

**Keywords:** host–pathogen interaction, iron sequestration, micronutrients, nutritional immunity, wound infection, zinc homeostasis

## Abstract

Nutritional immunity is an essential defense process by which the host restricts the supply of critical micronutrients to invading pathogens, thus hindering their survival and growth. During wound infections, this mechanism is instrumental in determining the course of host–pathogen interactions. This article discusses the pathophysiology of wound infection, beginning with the classification of wounds as acute and chronic wounds, and emphasizes how compromised epithelial barriers and chronic inflammation provide a niche for microbial colonization. The host immune response during the healing of wounds is tightly regulated, with inflammatory mediators, neutrophil infiltration, macrophage polarization, and eventual tissue regeneration. However, in the case of normal pathogens like *Staphylococcus aureus* , *Pseudomonas aeruginosa* , and *Escherichia coli* , they exploit the microenvironment within the wound for accessing necessary nutrients while avoiding immune detection. The premise for this is nutritional immunity, where the host withholds metals like iron, zinc, manganese, and copper from access to prevent nutrient delivery to pathogens. Pathogens retaliate by generating siderophores, transporters, and metallophores to enable nutrient capture. In addition, clinical use of metal‐chelating intelligent dressings, ionophore‐antibiotic hybrids, and metal modulation technologies has improved our capability to control infection via nutritional routes. At the same time, immunonutrition with such nutrients as omega‐3 fatty acids, arginine, glutamine, nucleotides, and antioxidant vitamins C and E has been demonstrated to have promise in promoting wound healing and maintaining immune resistance after surgery. This review emphasizes the necessity of targeting micronutrient pathways and incorporating nutritional immunity in wound care regimes for better clinical outcomes and prevention of infection‐related complications.

## Graphical abstract

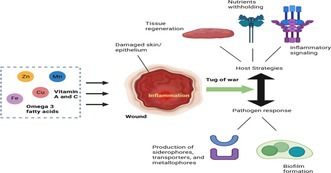

**Abbreviations**
CRP: C‐reactive protein
IL‐6: interleukin‐6
ROS: reactive oxygen species
TGF‐β: transforming growth factor‐beta
TNF‐α: tumor necrosis factor‐alpha
VEGF: vascular endothelial growth factor

## 1 Introduction

Wound infections are a serious public health issue with a significant impact on patient outcomes, quality of life, and healthcare expenses globally. A wound is infected when pathogenic microorganisms infect damaged tissue by breaching the initial immune defenses of the host and multiplying in the wound environment. Infections can arise in a great multitude of wound types, such as surgical cuts, traumatic wounds, burns, pressure sores, and diabetic foot ulcers. The existence of infection slows the healing process, induces chronic inflammation, and enhances the risk of systemic complications such as cellulitis, osteomyelitis, sepsis, and even mortality in extreme cases (Bowler et al. 2001; Leaper et al. 2004). The burden of wound infections is particularly significant in hospital‐acquired infections, where surgical site infections (SSIs) alone contributed to as much as 20% of all nosocomial infections. The pathogens most frequently cultured from infected wounds are *Staphylococcus aureus* (methicillin‐resistant *S. aureus* included), *Pseudomonas aeruginosa* , *Escherichia coli* , and *Enterococcus faecalis* (Lipsky et al. 2006). These pathogens not only cause tissue repair to be delayed by the production of toxins and biofilms but also resist immune detection and remain in the wound bed. Chronic wounds like venous leg ulcers and diabetic foot ulcers are particularly susceptible to polymicrobial colonization, where the interaction between aerobic and anaerobic microbes maintains a chronic inflammatory environment that is deleterious to healing (James et al. 2008). In addition, antibiotic resistance has made the problem more formidable, with conventional therapies not being able to clear infections, and alternative and integrative treatments that enhance host defense mechanisms with specific targeting of microbial growth becoming the need. Nutritional immunity is a host defense mechanism that entails sequestration of trace elements necessary for invading pathogens, for example, iron, zinc, and manganese, to restrict their growth and proliferation (Hood and Skaar 2012). The term was originally used to refer to the capacity of the body to withhold iron in the event of infection, realizing that pathogens need iron for critical biological processes such as respiration, DNA synthesis, and oxidative stress management. The host, therefore, limits iron availability by elevating iron‐binding proteins such as transferrin, lactoferrin, and ferritin in the circulation and at sites of infection (Soares and Weiss 2015). This is not specific to iron. There are similar tactics for other micronutrients. For example, the host uses calprotectin, a calcium‐ and zinc‐binding protein secreted by neutrophils, to sequester manganese and zinc, and this disrupts microbial enzymatic processes and microbial growth (Kehl‐Fie and Skaar 2010). These micronutrient‐withholding mechanisms are an integral part of innate immunity and work synergistically with inflammatory cytokines, phagocytic cells, and antimicrobial peptides to regulate pathogen dissemination. Nutrition has a basic function in regulating immune competence, affecting the host's resistance to infection and the ability of the pathogen to survive. Macronutrients (carbohydrates, fats, and proteins) and micronutrients (vitamins and minerals) are essential for immune competence and promoting tissue repair, leukocyte activation, and cytokine production (Calder 2020). Micronutrients, including iron, zinc, copper, selenium, and vitamins A, C, D, and E, have established roles in promoting both innate and adaptive immunity. Pathogens, conversely, are reliant on host‐derived nutrients for their survival and replication. The availability of iron and zinc is essential for bacterial biofilm development, oxidative stress resistance, and virulence gene activation (Zhang et al. 2024). The nutritional environment of the wound, thus, plays a critical role in dictating the pattern of microbial colonization, the severity of infection, and the direction of healing. The main goal of this study is to explore the interaction among nutritional status, trace element availability, and the outcome of wound infection.

## 2 Literature Search Strategy

A thorough review of the literature utilizing the Google Scholar, PubMed, Scopus, and Web of Science search engines served as the foundation for this narrative review. Peer‐reviewed publications released between January 2000 and December 2024 were included in the main search. Other seminal publications that were released prior to 2000 were also taken into consideration to offer foundational information when required. The following terms were utilized along with their combinations: micronutrients, nutrients, nutritional immunity, wound infection, wound healing, iron sequestration, manganese, copper, and zinc. Only English‐language publications were taken into consideration.

## 3 Pathophysiology of Wound Infection

### 3.1 Types and Classification of Wounds: Acute vs. Chronic

**FIGURE 1:**
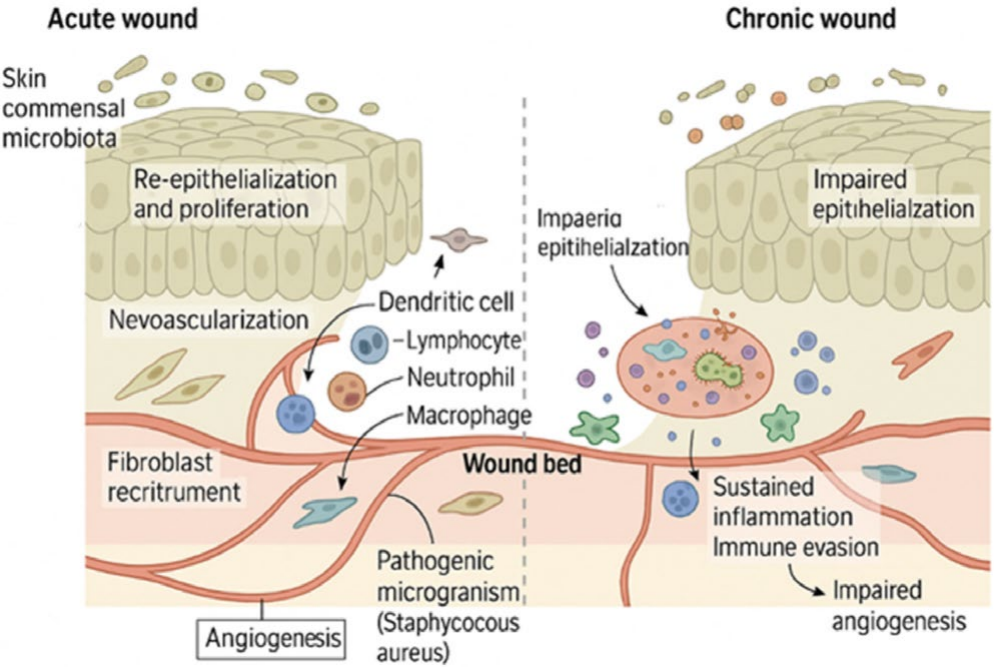
A contrast of the recuperation of acute and chronic wounds. Unlike chronic wounds, which are characterized by poor epithelialization and continuous swelling, acute wounds have structured immunological and vascular reactions. *Source:* Li et al. (2022).

Wounds are usually classified according to their course of healing, cause, and time required for healing. Acute wounds are those that have a normal and timely healing process, going through the stages of hemostasis, inflammation, proliferation, and remodeling without much interference. These are surgical wounds, lacerations, abrasions, burns, and traumatic wounds. In acute wounds, healing is generally achieved within a reproducible period, usually 4–6 weeks, as long as there is no underlying infection or pathology that would impede recovery (Hess 2011). However, chronic wounds have a chronic or arrested healing process, typically extending for weeks or even months. They occur due to a disturbance in one or more healing phases and are often seen in conjunction with an underlying condition like diabetes mellitus, peripheral vascular disease, venous insufficiency, or immunocompromised conditions (Hess 2011). Some of the common chronic wounds are pressure ulcers, diabetic foot ulcers, venous leg ulcers, and arterial ulcers. They all have pathological inflammation, ongoing infection, hypoxia, and defective cellular response. The chronicity of these injuries offers a very good setting for microbial colonization and infection, further promoting tissue destruction and prolonging healing (Edwards and Harding 2004). The categorization of wounds also comes in the aspect of the extent of contamination. Wounds may be classified as clean, clean‐contaminated, contaminated, or dirty/infected. Clean wounds refer to wounds that show no sign of infection or inflammation and entail sterile surgical wounds. Clean‐contaminated wounds can include surgical incursion into a body cavity with normal flora, for example, the gastrointestinal tract. Contaminated wounds have evidence of bacterial contamination, usually due to traumatic injury. Infected or dirty wounds are those with gross evidence of infection, such as purulence, erythema, warmth, and pain (Mangram et al. 1999). Knowledge of these classifications is important in determining treatment and predicting the risk of infection (Figure 1).

As highlighted in Figure 1, immune system cells, fibroblasts, and endothelial cells work in concert to facilitate blood vessel development, re‐epithelialization, and tissue regeneration in acute wounds. On the other hand, chronic wounds exhibit decreased angiogenesis, disturbed immunological modulation, and continuous microbial growth (e.g., *S. aureus* ). This imbalance results in a persistent wound setting and a delayed recovery.

### 3.2 Host Immune Response During Wound Healing

Host immunity plays a pivotal role in wound healing. Immediately upon injury, a highly sophisticated and complex immune reaction is triggered to prevent infection, remove cellular debris, and initiate healing of tissues. Both innate and adaptive immunity play a part, and the innate immunity serves as the first defense mechanism (Eming et al. 2007). The process is stimulated by hemostasis when the platelets coagulate and release the clotting factors to form a fibrin clot. Not only does the clot prevent further bleeding, but it also acts as an instrument of migration of cells along with a growth factor source, a provisional matrix for cell migration, and a source. There is the inflammatory phase afterward that begins with the recruitment of neutrophils and macrophages to the wound site. Neutrophils are the first to arrive, releasing reactive oxygen species (ROS) and proteolytic enzymes to kill invading pathogens and debride dead tissue. Excessive neutrophil activity, however, will cause damage to tissues and delayed healing (Schultz et al. 2011). Macrophages, which arrive after neutrophils, execute phagocytosis of the invading pathogens and apoptotic cells and secrete cytokines and growth factors like transforming growth factor‐beta (TGF‐β), vascular endothelial growth factor (VEGF), and tumor necrosis factor‐alpha (TNF‐α). These mediators control inflammation, induce angiogenesis, and cause fibroblast and keratinocyte recruitment for tissue healing (Wlaschek and Scharffetter‐Kochanek 2005). The immune response in chronic wounds becomes disordered. Chronic inflammation, macrophage polarization defects, and breakdown of the MMP‐TIMP balance cause extracellular matrix component breakdown and re‐epithelialization defects (Theocharidis et al. 2020). Adaptive immunity plays a role in long‐term monitoring of the wound. T lymphocytes can control the immune microenvironment by secreting cytokines, while B cells release antibodies that help in the neutralization of the pathogen. Adaptive immune responses can be deficient in chronic wounds, particularly among immunocompromised individuals, and opportunistic infections can take hold and become established (MacLeod and Mansbridge 2016).

### 3.3 Common Pathogens Associated With Wound Infections

**FIGURE 2:**
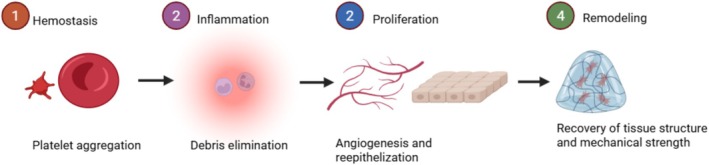
Stages of the recuperation process of wounds. In order to regain tissue integrity, the process consists of four overlapping steps: hemostasis, swelling, proliferation, and remodeling with distinct cellular activity. This figure was generated by using biorender.

Wound infections can be caused by a wide variety of microorganisms, including bacteria, fungi, and, less commonly, viruses. The most frequently implicated pathogens are bacterial; however, the nature of the infecting organisms depends on variables like wound type, site, patient's immune status, and environmental exposure. Among bacterial pathogens, *S. aureus* is the most commonly cultured organism in both acute and chronic wounds, and methicillin‐resistant *S. aureus* (MRSA) is another challenge, as it is resistant to over one antibiotic. *S. aureus* harbors an assortment of virulence factors, including surface adhesins, toxins, and enzymes involved in tissue invasion and immune evasion (Tong et al. 2015). *P. aeruginosa* is an opportunistic pathogen, particularly in burn and chronic wounds. It grows optimally under moist conditions and is inherently resistant to most antibiotics. *P. aeruginosa* produces biofilms—organized groups of bacteria embedded with an extracellular matrix of protective molecules—resulting in chronic infection and impaired wound healing (Fazli et al. 2014). Other significant pathogens are *E. coli* , *Klebsiella pneumoniae* , *Proteus mirabilis* , *Enterococcus faecalis* , and anaerobes like *Bacteroides fragilis* . Polymicrobial infections that include anaerobes and aerobic bacteria co‐exist in the majority of chronic wounds, forming a sophisticated microbial community hard to treat (Dowd et al. 2008). Fungal infection of the wound, although less common, takes place in immunocompromised individuals or on prolonged antibiotic therapy. Filamentous molds such as Aspergillus and Candida infect and colonize wounds, particularly in the in‐hospital setting. Fungal infection is a poor prognostic indicator and usually necessitates systemic antifungal therapy (Alonso‐Monge et al. 2021) (Figure 2).

In order to stop blood loss, hemostasis starts the platelet aggregation that heals wounds (see Figure 2). Inflammation follows, during which phagocytes and neutrophils eliminate debris and infectious agents. Fibroblasts stimulate angiogenesis while keratinocytes participate in re‐epithelialization throughout the proliferation period. Fibroblasts and other cells eventually recover tissue structure and mechanical durability during the step of remodeling.

### 3.4 Mechanisms of Pathogen Survival and Proliferation in Wound Sites

**FIGURE 3:**
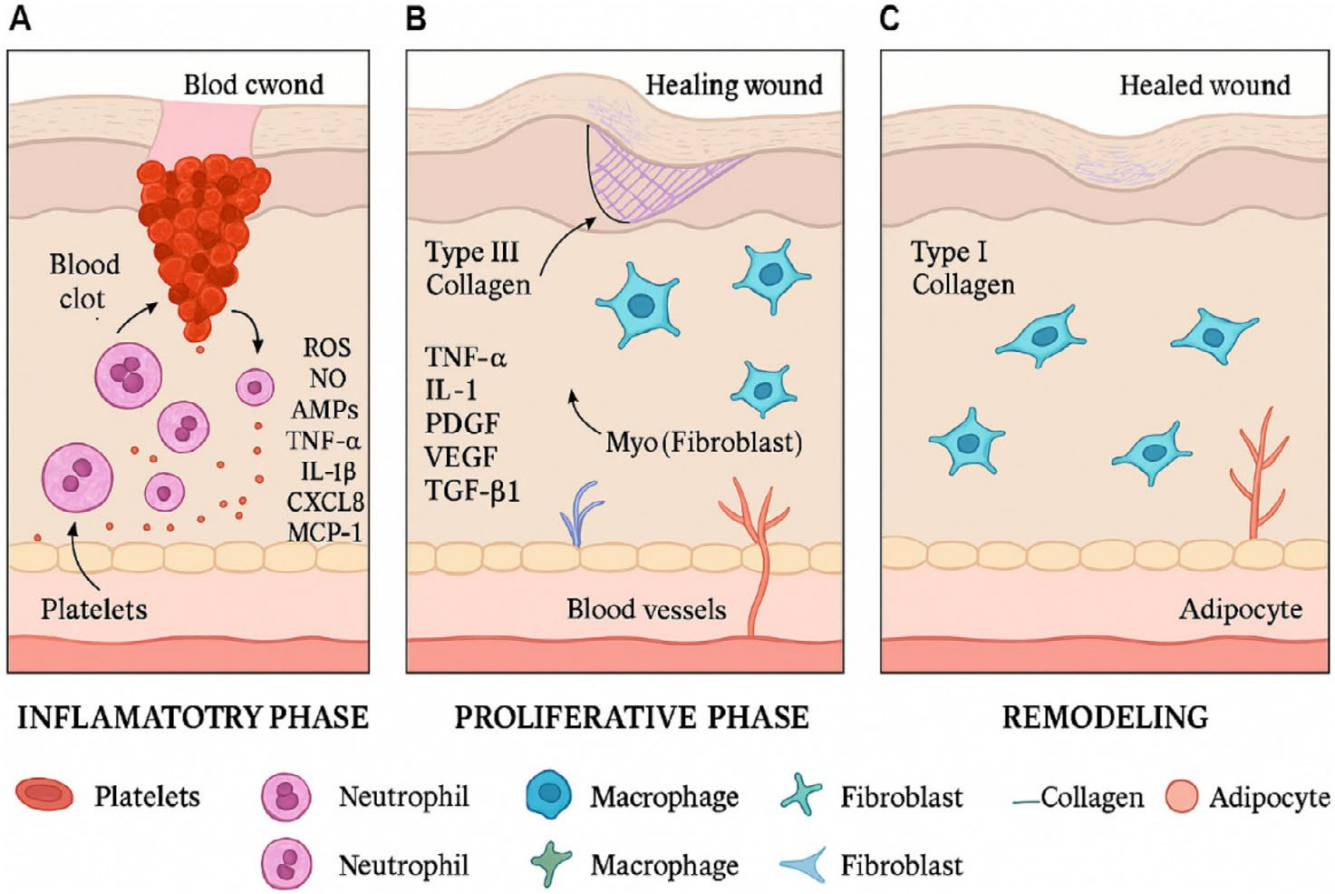
Cellular and molecular reactions occur during the various stages of the healing process. With a focus on vital immune cells, fibroblasts, and collagen buildup responsible for tissue regeneration, the image illustrates the inflammatory, proliferative, and restructuring steps. *Source:* Jakovija and Chtanova (2023).

When pathogens infect wounds, survival and proliferation are facilitated by multiple adaptive mechanisms that allow them to survive host defense mechanisms as well as antimicrobial therapy. Maybe one of the most widely used survival processes among bacteria, especially in chronic wounds, is the biofilm model. Biofilms are structured microbial communities in contact with surfaces and a self‐secreted extracellular polymeric material (EPS) consisting of polysaccharides, proteins, and nucleic acids. The biofilm matrix protects the bacteria from phagocytosis, antibody penetration, and antibiotic action. Bacteria in biofilms exhibit altered phenotypes, like reduced metabolic activity and increased expression of resistance genes. The presence of biofilms in wounds is associated with chronic infection, delayed healing, and recurrence risk (Franklin et al. 2015). One essential mechanism is the secretion of virulence factors that help in colonization, tissue invasion, and immune evasion. For instance, *S. aureus* secretes protein A, which binds to the Fc region of immunoglobulin G, inhibiting opsonization and phagocytosis. *S. aureus* also secretes hemolysins and leukocidins that lyse host cells and inhibit immune responses. *P. aeruginosa* secretes elastases, exotoxins, and pyocyanin, a pigment that generates ROS and inhibits immune cell function (Lau et al. 2004). Certain bacteria can survive intracellularly, evading immune detection and antibiotic treatment. *S. aureus* , for instance, can infect keratinocytes and endothelial cells, creating intracellular reservoirs that lead to recurrent infections (Gresham et al. 2000). Other pathogens become resistant to antimicrobial peptides and antibiotics via genetic mutations, efflux pumps, and horizontal gene transfer (Poole 2004). The pathogen's survival is also facilitated by the wound microenvironment itself. Hypoxia, low pH, and exudate overload are common in chronic wounds and can impair immune cell function, as well as provide niches for anaerobic and facultative anaerobic organisms. Degradation of the extracellular matrix by MMPs and the presence of necrotic tissue also provide a basis for microbial colonization and biofilm development (James et al. 2008). Moreover, the host's nutritional state, blood glucose, and comorbidities have a great impact on microbial growth. For instance, in diabetic individuals, hyperglycemia affects neutrophil function, diminishes chemotaxis and phagocytosis, and impairs tissue perfusion, all of which predispose to increased infection risk and impaired healing (Marhoffer et al. 1992). In summary, the pathophysiology of wound infection is an intertwined phenomenon between wound, host defense, microbial virulence, and external surroundings. Acute and chronic wounds are characterized by great differences in their infectability and potential for healing. The immune system is vital to wound healing, but when its regulation is deranged in the case of chronic wounds, it creates a conducive environment for colonization by microbes. Multiple pathogens, with *S. aureus* and *P. aeruginosa* leading the pack, cause wound infection, each possessing advanced mechanisms of evading host immunity and persisting in antagonistic wound microenvironments. Biofilm formation, virulence factor secretion, intracellular persistence, and mechanisms of resistance are all factors responsible for the severity and chronic nature of infected wounds. Wound infections, therefore, need a multi‐disciplinary treatment approach with the use of proper wound management, specific antimicrobial therapy, support to the immune system, and sophisticated diagnostic methods to improve the healing process and minimize complications (Figure 3; Table 1).

**TABLE 1:** Nutritional interventions in wound healing.

Rationale	Treatment Used	Objective	Outcomes	References
Explore the role of micronutrients in immune function	Micronutrient supplementation	To examine the influence of micronutrients on immune function and wound healing	Micronutrients (e.g., zinc, vitamin A, C) play a critical role in supporting immune function and healing	Pecora et al. (2020)
Investigate the effects of polyunsaturated fatty acids (PUFAs) on inflammation	Omega‐3 and omega‐6 PUFAs	To assess the modulation of inflammatory responses by different fatty acids	Omega‐3 PUFAs significantly reduce inflammation, contributing to improved healing in wounds	Calder (2020)
Assess acute‐phase proteins in response to inflammation	No specific treatment (focus on inflammatory markers)	To understand the role of acute‐phase proteins in inflammatory response and wound infection	Elevated acute‐phase proteins (e.g., C‐reactive protein) indicate a systemic inflammatory response	Sproston and Ashworth (2018)
Examine nutritional modulation of immune responses in wound healing	Nutritional interventions (vitamins, minerals)	To explore the role of diet in modulating immune responses during wound healing	Nutrients such as vitamin A and C are essential for proper immune response and wound repair	Santo et al. (2024)
Investigate zinc's role in immune function and wound healing	Zinc supplementation	To explore the immune‐boosting effects of zinc and its involvement in wound healing	Zinc supplementation improves immune function and accelerates wound healing in deficient individuals	Lin et al. (2017)
Evaluate the effect of micronutrients on immune function in chronic wounds	Micronutrient supplementation	To determine how micronutrients support immune function in chronic wound patients	Micronutrients like vitamins C, E, and zinc significantly aid in the healing of chronic wounds	Seth et al. (2024)
Investigate the impact of micronutrient deficiencies on wound healing	Vitamin and mineral supplementation	To understand how deficiencies in vitamins and minerals affect wound healing	Deficiencies in micronutrients such as vitamin A, C, and zinc delay wound healing and immune response	Ju et al. (2023)
Examine the effects of micronutrient supplementation in chronic wounds	Micronutrient supplementation	To assess the role of micronutrients in the healing of chronic wounds and their immune modulation	Supplementation enhances wound healing, especially in patients with nutritional deficiencies	Cereda et al. (2015)
Investigate the modulation of immune responses by vitamin D.	Vitamin D supplementation	To study the effect of vitamin D on immune function and wound healing	Vitamin D enhances immune response, playing a vital role in managing infections and wound repair	Wu et al. (2024)
Assess the role of vitamins and trace elements in wound infection	Vitamins (A, C, D), trace elements (zinc, iron)	To explore the relationship between micronutrients and infection resistance in wound healing	Micronutrients such as vitamin C and zinc help reduce the risk of wound infections and promote healing	Karunakaran et al. (2025)
Investigate vitamin D's role in immune modulation during wound healing	Vitamin D supplementation	To determine how vitamin D influences immune responses and wound healing	Vitamin D supplementation improves immune function and accelerates wound healing	Siregar and Hidayat (2023)
Examine host‐pathogen interactions and their impact on wound healing	No specific treatment (focus on immune interactions)	To explore how the immune system and pathogens influence wound healing outcomes	Host immune responses and pathogen virulence factors significantly affect wound infection and healing	Trøstrup et al. (2020)
Investigate the effect of zinc and vitamin C supplementation in wound infections	Zinc and vitamin C supplementation	To assess the effects of zinc and vitamin C on wound infection management	Zinc and vitamin C improve immune responses and infection management in wound healing	Saeg et al. (2021)
Study iron metabolism and its impact on wound healing and infection	Iron supplementation	To evaluate the role of iron in wound healing and infection management	Iron supplementation aids in wound healing by improving oxygenation and immune response	Boncheva (2023)
Investigate the role of amino acids in immune modulation and wound healing	Amino acid supplementation	To explore how amino acids influence immune function and wound healing	Amino acids promote collagen formation and immune modulation, accelerating wound healing	Arribas‐López et al. (2021)
Study the effects of nutrition on wound healing and infection	Nutritional therapy (vitamin/mineral supplements)	To understand how nutrition impacts the healing of wounds and infections	Adequate nutrition enhances wound healing, reduces infection rates, and supports immune function	Daher et al. (2022)
Explore the role of trace elements and vitamins in wound healing	Trace element and vitamin supplementation	To investigate how vitamins and trace elements aid in wound healing and immune function	Trace elements and vitamins, especially vitamin C and zinc, are crucial for optimal wound healing	Penny et al. (2022)
Assess the role of vitamin C in immune function and wound healing	Vitamin C supplementation	To determine the immune‐boosting effects of vitamin C in wound healing	Vitamin C supplementation enhances collagen synthesis, immune response, and wound healing	Bechara et al. (2022)
Investigate omega‐3 fatty acids’ role in immune modulation and wound healing	Omega‐3 fatty acids	To explore the impact of omega‐3 fatty acids on immune responses and wound repair	Omega‐3 fatty acids modulate immune responses, reducing inflammation and promoting healing	Serini and Calviello (2021)
Study the role of microbiota in immune regulation during wound infection	No specific treatment (focus on microbiota)	To explore how the microbiota influences wound infection and immune responses	Microbiota composition significantly influences immune regulation and healing in infected wounds	Zielińska et al. (2023)

The inflammatory stage, as seen in Figure 3, includes neutrophil/macrophage stimulation and accumulation of platelets, which generates cytokines, chemokines, and ROS.

In the growing stage, fibroblasts and myofibroblasts use growth regulators including VEGF and TGF‐β1 to promote angiogenesis, collagen type III generation, and wound closing.

When type I collagen takes the place of type III collagen during the remodeling stage, the recovery process is completed and elasticity is restored.

Table 1 summarizes multiple research findings that, in tandem, demonstrate the essential part that nutritional supplements—specifically, micro nutrients, vitamins, trace minerals, amino acids, and fatty acids—play in boosting wound repair and the immune system's performance. Vitamins A, C, D, E, and zinc regularly show up as essential elements for decreasing inflammation, boosting collagen formation, and regulating both innate and adaptive immune responses across a variety of therapies. Vitamin D's immune‐regulating capacity is notably highlighted in a number of investigations, especially in relation to improving macrophage activity and the synthesis of antimicrobial peptides. It has also been demonstrated that omega‐3 fatty acids reduce pro‐inflammatory mediators, which is favorable for chronic wounds. Although most research focused on individual dietary nutrients, certain investigations backed a synergistic model in which coupled micronutrient mixtures (such as vitamins C and E with zinc or iron) had more noticeable effects, especially in communities with pre‐existing deficiencies. This body of research supports the beneficial effects of immunonutrition as a supplement to wound care techniques, especially for individuals with persistent wounds or those who are at nutritional vulnerability.

## 4 Nutritional Immunity: An Overview

In the 1940s, Schade and Caroline (1944 and 1946) identified transferrin, an iron‐binding protein found in human plasma and egg whites, which helped to clarify the connection between trace minerals and immunity (Schade and Caroline 1944, 1946). Iron was discovered to be bound by transferrin, which prevented microbial development and sequestered iron from pathogens. This approach was dubbed nutritional immunity by Weinberg (1975). All life depends on transition metals, which invasive microbial pathogens must obtain in order to proliferate within the host and spread infection. In order to fight this, the host takes advantage of the toxicity and necessity of food metals by creating variables that restrict metal availability. This causes infections to either starve or accumulate too much metal to intoxicate them, a mechanism known as “nutritional immunity” (Healy et al. 2021).

The host's ability to sequester bioavailable trace metals like iron, zinc, and copper in order to reduce the pathogenicity of invasive pathogens is known as nutritional immunity. Restricting the absorption of free trace metals by immune system cells is one of the most conserved functions of the innate immune system. This function not only hides these essential nutrients from invasive bacteria but also tightly controls host immune cell responses and function (Lopez and Skaar 2018). A panel of host scavengers that have a strong affinity for metal ions controls this process, allowing them to effectively alter metal concentrations in reaction to microbial assaults. Numerous proteic factors, such as the well‐known hepcidin, lactoferrin, siderocalin, metallothionein, and calprotectin, are involved in regulating metal homeostasis in the host. These proteins have the dual roles of protecting host cells, transporting minerals, and preventing pathogens from accessing minerals (Healy et al. 2021). Because they control the availability of vital micronutrients, innate immune cells like neutrophils and macrophages are crucial for nutritional immunity. By releasing metal‐sequestering proteins like lactoferrin and calprotectin, these cells provide an environment deficient in nutrients that prevents the formation of pathogens (Monteith and Skaar 2021; Marchetti et al. 2019).

## 5 Micronutrients and Host–Pathogen Competition in Wounds

Micronutrients are important in host–pathogen competition because both the pathogen and the host rely on them for survival and function.

### 5.1 Iron

One of the most prevalent elements on the crust of the earth is iron, a micronutrient that is vital to most living things. For their cells to survive and proliferate during an infection, the pathogen and the host must continue to have access to iron. Thus, a key element of the host's innate immune response during infection is restricting the iron availability of invasive microorganisms. Iron functions as a redox catalyst that either gives or receives electrons since it occurs in two oxidation states: ferrous cation (Fe^2+^) and ferric cation (Fe^3+^) (Ashraf et al. 2024). Iron's redox potential makes it easily accessible for usage in a variety of biological functions, but at high concentrations, it may also cause toxicity and cell death by catalyzing the Fenton reaction, which releases free hydroxyl radicals that harm proteins, lipids, and DNA. Thus, to guarantee iron's vital nutritional benefits and avoid its harmful consequences, its acquisition, transportation, usage, and storage must be tightly controlled through a precisely calibrated process (Rodríguez‐García et al. 2021). Proteins like ferritin, hepcidin, hemoglobin, lactoferrin, transferrin, and calprotectin bind iron and decrease its accessibility in the outside environment, thereby mediating iron sequestration. In addition to its role in innate immune responses, transferrin is a crucial component of iron homeostasis. The four forms of transferrin found in mammals are the molecules that inhibit the activity of carbonic anhydrase, melanotransferrin, lactoferrin, and serum transferrin. Iron sequestration from invasive pathogens is one way that serum transferrin and lactoferrin contribute to nutritional immunity. Because transferrin has a high affinity for iron, it can keep the amount of free iron in bodily fluids low, which stops invasive bacteria from having access to iron (Rodríguez‐García et al. 2021; Barber and Elde 2014). A member of the transferrin family, lactoferrin (Lf) is an 80 kDa glycoprotein that binds iron. It is a cell‐secreted molecule that connects the innate and adaptive immune systems in mammals and is present in bovine milk as well as other exocrine secretions such as bile, saliva, and lacrimal fluid. In addition to being antiviral and antibacterial, it possesses significant immunological qualities. Lactoferrin's capacity to bind to iron and render it inaccessible to bacteria is primarily responsible for its antibacterial properties (Ashraf et al. 2024). According to García‐Montoya et al. (2012), its methods of action include interactions with the molecular and cellular components of both hosts and pathogens in addition to its ability to bind to iron. The iron‐regulatory hormone hepcidin modulates both the total amount of iron in the body and circulating iron concentrations. The cellular iron exporter ferroportin, which transports iron to plasma from intestinal iron absorption and iron storage, is regulated by hepcidin, which is released by hepatocytes (Nemeth and Ganz 2023). By controlling iron metabolism, hepcidin may have a significant role in immunological modulation, inflammatory disorders, and cancer (Singh et al. 2011). Being an acute‐phase reactant, ferritin is regarded as a pathogen‐proof method of storing iron. It stores extra iron in a form that is not redox active (Kotla et al. 2022). Ferritin limits pathogens' capacity to spread infection by binding and sequestering excess iron, preventing them from gaining access to this vital nutrient (Gehrer et al. 2022). At infection sites, neutrophils and epithelial cells release the abundant antibacterial protein calprotectin (CP). It is essential for the host's defense against bacterial and fungal infections such as Aspergillus fumigates, *Candida albicans* , and *S. aureus* . According to reports, calprotectin (CP) withheld iron from *P. aeruginosa* by using the His6 site (Zygiel and Nolan 2019). Heme is necessary for hemoproteins to operate, which include detoxifying host immunological effectors and generating energy through the electron transport chain, among other functions. The main source of bioavailable iron is heme and hemoproteins. Although host heme is sequestered in high‐affinity hemoproteins, bacterial pathogens must either synthesize heme or obtain it from the host during infection (Choby and Skaar 2016). Therefore, bacterial pathogens face another obstacle in gaining possession of host iron: the sequestration of iron or heme from host proteins. Ferroportin (FPN), an iron transport protein that releases iron from the cytoplasm to the extrinsic environment, is expressed by macrophages, which additionally perform a crucial role in regulating iron availability. According to a study, FPN is quickly removed from the phagosomal barrier by macrophages, which stops Fe from extruding into the phagosome lumen. This might contribute to the expanding list of host mechanisms of nutritional immunity by further limiting the uptake of iron by bacteria (Flannagan et al. 2021). Because iron is essential for both the host and the pathogen, the two are in competition for it. In the meantime, pathogens use a variety of iron acquisition strategies, including siderophores and specific receptors, to extract iron from the host.

**FIGURE 4:**
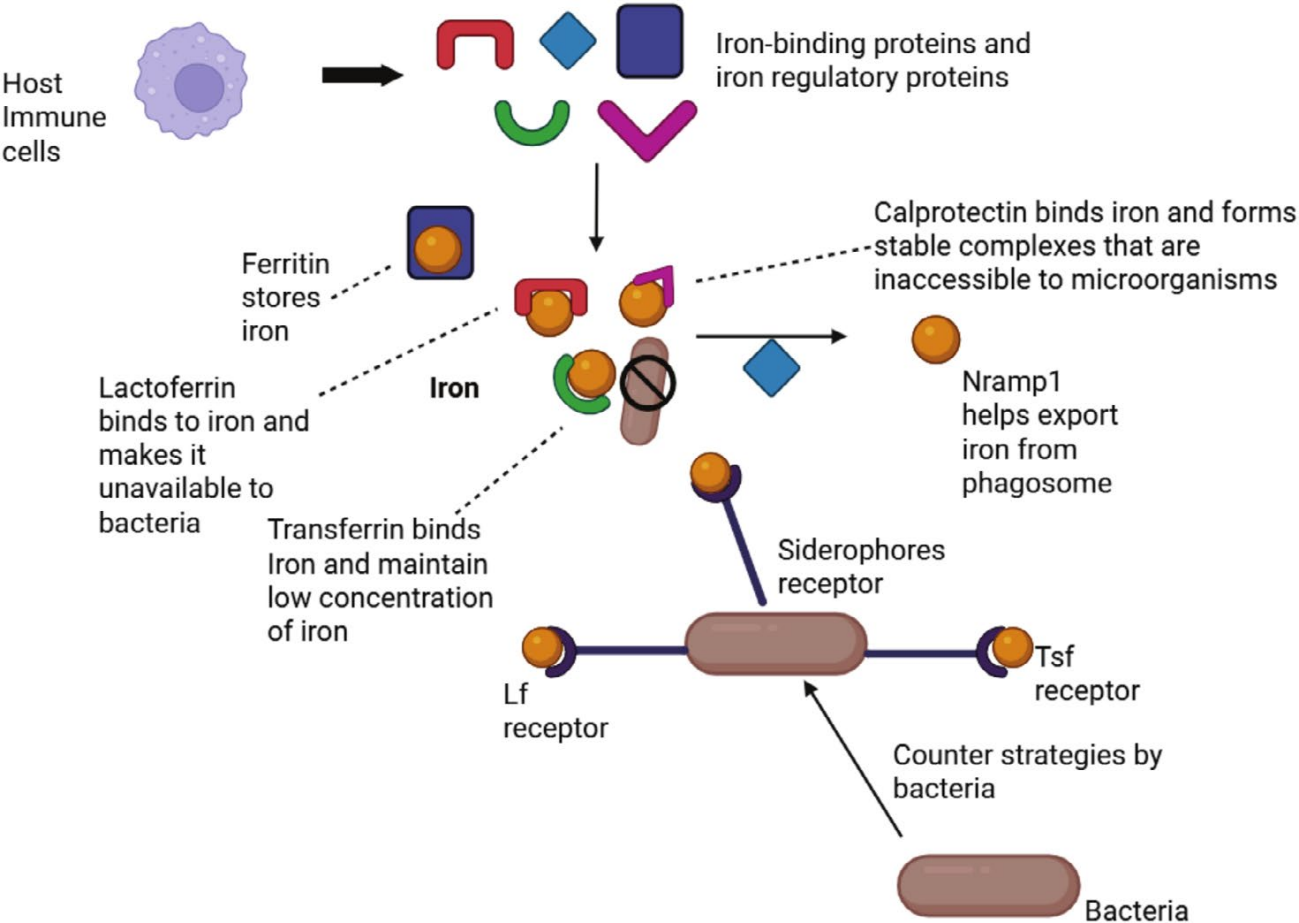
Host and pathogen mechanisms for iron sequestration. The host restricts bacterial utilization of iron by using transporters (like Nramp1) and metal‐binding proteins (including transferrin, lactoferrin, ferritin, and calprotectin), whereas pathogens exhibit siderophores and receptors to navigate around these protections. This figure was generated by utilizing biorender.

The two main siderophores that *P. aeruginosa* synthesize to get iron are pyoverdine (PVD) and pyochelin (PCH). According to Braud et al. (2010) and Schalk and Perraud (2023), both siderophores have the capacity to chelate a wide range of other metals in addition to iron. The main heme–iron absorption pathway used by the opportunistic pathogen *S. aureus* is the iron‐responsive surface determinant (Isd) system (Hammer and Skaar 2011). Additionally, it employs siderophores, which are high‐affinity iron chelators. Two staphyloferrins (siderophores) are produced and secreted into the extracellular environment by *S. aureus* in order to scavenge iron (Conroy et al. 2019). Siderophores can extract the vital metal from the host iron‐binding proteins because of their strong affinity for iron (Figure 4).

As seen in Figure 4, ferritin accumulates internal iron, Nramp1 releases it from phagosomes, and host immune system cells use proteins like transferrin and lactoferrin to attract external iron.

Moreover, calprotectin holds iron to stop microbes from utilizing it. To seek out iron from host‐bound sites and enable survivability and growth regardless of host defenses, pathogens respond by releasing siderophores and expressing receptors.

### 5.2 Zinc

Zinc is probably the transition metal that is utilized in a greater number of proteins as a structural or catalytic cofactor. It has a role in controlling inflammatory reactions and is necessary for immune cells like T lymphocytes and macrophages to operate properly. One‐third of people worldwide suffer from zinc deficiency, which is linked to a higher risk of contracting bacterial infections (Eijkelkamp et al. 2019). One of the important participants in host–pathogen interactions has long been thought to be zinc (Du Pré et al. 2022). In order to strengthen the immune system and stop the pathogen from growing and infecting others, the host sequesters zinc after infection. To prevent bacteria from acquiring too much zinc, some zinc‐sequestering proteins are produced and drawn to the infection site. For example, Zrt/Irt‐like protein (ZIP)/ZnT family proteins and other zinc storage proteins allow the host to control zinc levels. One of the 14 members of the ZIP family, the human zinc transporter Zrt/Irt‐like protein 8 (ZIP8), is particularly crucial for the flow of divalent cations, such as zinc, into the cytoplasm of macrophages. Additionally, it has been seen to reside on cellular organelle membranes, where it can act as an efflux pump to move zinc into the cytosol (Pyle et al. 2017). Additionally, calprotectin contributes to the sequestration of this vital micronutrient metal, reducing the amount of it that microorganisms may access (Liu et al. 2012). According to Kehl‐Fie et al. (2011), calprotectin sequesters zinc and manganese, which improves neutrophil killing of *S. aureus* . However, zinc is essential to the survival and pathogenicity of pathogens. In order to survive in situations with low zinc levels, many infections have evolved high‐affinity zinc transporters that take up zinc from the host. Under such circumstances, Salmonella maximizes zinc availability by taking use of the ZnuABC zinc transporter (Ammendola et al. 2007). According to a study, Salmonella Typhimurium expresses a high‐affinity zinc transporter (ZnuABC) that enables it to evade calprotectin‐mediated zinc chelation. In response, pathogens compete with the host for zinc by using high‐affinity Zn^2+^ transporters, including ZnuACB (Xia et al. 2021; Bobrov et al. 2014). The bacterial synthesis of tiny molecules called zincophores is another way that bacteria obtain zinc. For instance, the metal‐binding small molecule staphylopine, which facilitates zinc acquisition, is produced by *S. aureus* (Lonergan and Skaar 2019).

### 5.3 Manganese

Another vital micronutrient that plays a role in tissue healing, antioxidant defense, and enzyme function is manganese. Because manganese shortage affects collagen formation and immunological function, it can hinder wound healing. The host imposes manganese restriction through a number of methods during infection. These include sequestering extracellular manganese, removing manganese from the phagolysosome, and using other metals to stop bacteria from acquiring manganese (Juttukonda and Skaar 2015). There is growing evidence that calprotectin (CP) can affect the fight for each one of these metal ions at the host–pathogen interface. Studies have revealed that CP has incredibly tight binding affinities for a variety of metals, including iron, zinc, and manganese—all of which are vital in biology (Murdoch and Skaar 2022). In the extracellular environment, calprotectin binds manganese, which is crucial for protection against germs like *S. aureus* . According to a study, CP sequesters Mn^2+^ via a special six‐histidine binding site, hence inhibiting bacterial growth (Damo et al. 2013). By sequestering manganese, calprotectin improves neutrophil killing of *S. aureus* , as demonstrated by Kehl‐Fie et al. (2011). It is believed that the Nramp/Slc11 family fights infection by preventing microorganisms from accessing iron, manganese, and potentially other metals. According to studies, Nramp1 may limit pathogen growth by reducing the availability of vital nutritional metals by transporting them out of the phagosome (Wessling‐Resnick 2015; Juttukonda and Skaar 2015). In addition to zinc, ZIP8 has been demonstrated to effectively transfer Mn into the cytosol of a range of host defense‐related cells (Fujishiro et al. 2012, 2011). Microorganisms use high‐affinity transporters to compete with host metal‐binding proteins in order to get around this nutritional constraint. Mn is acquired by *S. aureus* through the expression of the metal transporters MntABC and MntH. MntABC and MntH allow *S. aureus* to proliferate and retain manganese‐dependent superoxide dismutase function by competing with calprotectin for manganese (Kehl‐Fie et al. 2013; Radin et al. 2019). Superoxide dismutase (SOD) is one of the enzymes that *S. aureus* expresses and that can work with different metals. For instance, *S. aureus* SodM can use either iron or manganese, which enables the bacterium to produce infection and adapt to metal‐limited conditions (Garcia et al. 2017).

### 5.4 Copper

Another trace element that is essential to an organism is copper. As a catalytic cofactor for a variety of enzymes, copper's redox characteristics allow it to bind to lower molecular components and complex with proteins through complexation with cysteine, histidine, and methionine. The development and operation of the immune system are significantly impacted by copper deficiency. These effects include lowered neutrophil counts and functioning, reduced splenocyte growth, declined macrophage activity in fighting bacteria, decreased host susceptibility to various pathogens, compromised B cell antibody generation, and impeded cytotoxic T lymphocyte and helper T cell performance (Cheng et al. 2022). Cu can be sequestered by S100 proteins like Calprotectin (CP), which binds Cu(II) with subpicomolar affinity (Besold et al. 2018). While the sequestration of metals from bacteria has historically been included in the idea of nutritional immunity, the innate and adaptive immune systems can also actively use metals like copper to support bactericidal function (Ladomersky and Petris 2015). To prevent bacterial growth and multiplication, the host uses toxicity mechanisms that pump copper into the infected phagosome through its transporters (ATPase and Ctr1). Bacteria use copper tolerance genes to increase their pathogenicity within the host in order to overcome these protections (Ladomersky and Petris 2015). Certain bacterial pathogens become less virulent when copper homeostasis is disturbed. In order to produce an environment that supports their development and survival, pathogens can alter the host's copper homeostasis (Giachino and Waldron 2020) (Table 2).

**TABLE 2:** Micronutrients and their role in nutritional immunity.

Micronutrient	Influence in immune response	Mechanisms of sequestration	Pathogen response	Impact of deficiency	References
Iron	Essential for enzyme function and oxygen transport	Hepcidin, ferritin, lactoferrin	Siderophores, heme acquisition	Impaired immunological response and prolonged recovery	Ward et al. (2011)
Zinc	Important for protein structure and immune functioning	ZIP8, calprotectin, metallothioneins	High‐affinity zinc transporters, zincophores	Weakened immune system and elevated danger of infection	Samuelson et al. (2022)
Copper	Antioxidant defense and enzyme function	Calprotectin, Ctr1, ATPase	Copper homeostasis, copper tolerance genes	Higher susceptibility to infections and oxidative damage	Giachino and Waldron (2020)
Manganese	Metabolism and antioxidant defense	Calprotectin, NRAMP1, ZIP8	Manganese import proteins	Delayed recovery and compromised regeneration of tissue	Damo et al. (2013)

## 6 Clinical and Therapeutic Implication

### 6.1 Metal Modulation Capable Smart Dressings

**FIGURE 5:**
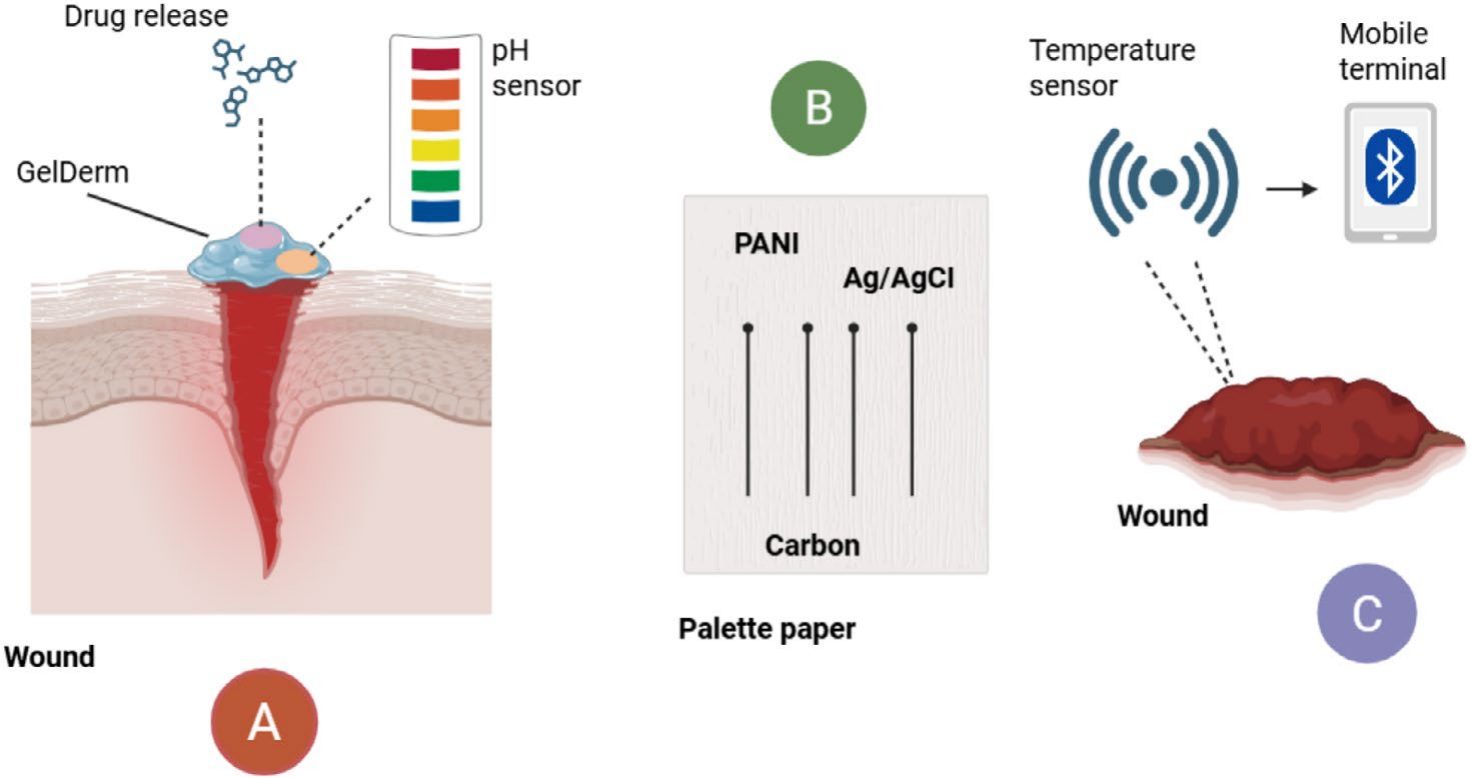
Multipurpose smart wound dressings that incorporate curative and detecting properties. Aspects of sophisticated dressings that can change pH (A), conduct electricity (B), and regulate temperature wirelessly (C). This figure was created by using biorender.

Developments in biomaterials have given rise to the development of intelligent wound dressings that can regulate metal ion concentrations at the wound site. The dressings can release or sequester metal ions such as zinc and copper, which play a crucial role in different phases of wound healing. Some smart dressings, for example, incorporate sensors that identify the wound status and deliver drugs upon exposure to some stimulants. Such technologies aim to amplify tissue regeneration, reduce infection incidence, and provide the best conditions for healing. The research continues to drive the biocompatibility and sensibility of the dressings toward clinical use (Li et al. 2024) (Figure 5).

As indicated by Figure 5, panel (A) demonstrates GelDerm, a pH‐responsive gel dressing that keeps the healing alkaline environment stable. Panel (B) shows a conductive dressing for electrochemical detection and activation made on a palette sheet with polyaniline (PANI), carbon, and Ag/AgCl.

Panel (C) shows an advanced dressing that allows for real‐time wound surveillance using a portable terminal. It has a temperature probe built on PDMS and Bluetooth dialog.

### 6.2 Immunonutrition Function in Postoperative Recuperation

Postoperative inflammatory reactions are exacerbated by surgical stress, which can impair immune function and make a person more vulnerable to infections. Additionally, this stress may hasten the loss of vital nutrients that are important in immunological control, which might result in shortages (Jabłońska and Mrowiec 2020). Patients undergoing pancreaticoduodenectomy received perioperative immunonutrition in a prospective randomized study, which had a positive impact on important immunological parameters such as T helper type 1 (Th1), Th2 cells, and CD4 (+) helper T (Th17) cells that produce interleukin (IL)‐17. Reductions in immunosuppression and postoperative infection problems were also noted (Suzuki et al. 2010). Preoperative immunonutritional supplementation was observed to lower IL‐6 levels in patients with a low skeletal muscle index having pancreaticoduodenectomy, and this was associated with a decrease in postoperative problems (Furukawa et al. 2021). Perioperative immunonutrition, which is administered both before and after surgery, was found to be more beneficial in lowering infection complications than immunonutrition administered either preoperatively or postoperatively in a meta‐analysis of patients having pancreaticoduodenectomy (Wang et al. 2021). Preoperative immunonutrition decreased the likelihood of infection complications by almost 50% when compared to the control group, according to a meta‐analysis of seven RCTs that included patients having gastrointestinal surgery. Furthermore, the immunonutrition group's average hospital stay was considerably shorter than the control group's, ranging from 15.3 to 13.6 days (Burden et al. 2012). Immunonutrition significantly reduced all postoperative problems, according to a meta‐analysis of 24 trials involving patients having surgery for gastrointestinal or head and neck cancer (Matsui et al. 2024). Immunonutrition has been shown to reduce infectious complications in patients with colorectal and stomach malignancies, according to meta‐analyses of many RCTs (Song et al. 2017; Xu et al. 2018). Immunonutrition also dramatically decreased the length of hospital stay by 34% and infection complications by 70.1%, according to an observational, retrospective cohort study of patients having gastrectomy who received nutritional assistance for an average of 10 days before and after surgery (Martínez González et al. 2024). Although it is difficult to draw firm conclusions about perioperative immunonutrition due to differences in study scale and outcome measures, it can be concluded that immunonutrition is essential for reducing immunosuppression and inflammation brought on by surgery, which may shorten hospital stays and prevent infectious complications.

#### 6.2.1 Omega‐3 FAs

According to a meta‐analysis, giving omega‐3 FAs continuously during the preoperative and postoperative phases of liver surgery was substantially more successful than giving them only during the preoperative or postoperative phases in lowering the rates of postoperative infections. Nevertheless, no advantages were noted in terms of ileus or mortality (Xiao et al. 2021). When omega‐3 FAs were administered to patients having hepatectomy, a randomized controlled study (RCT) indicated that the length of hospital stay was shortened and problems were lessened (Gong et al. 2016). On the other hand, a comprehensive review concluded that there was not enough evidence to warrant the administration of omega‐3 FAs before major gastrointestinal surgery (George et al. 2023). According to many meta‐analyses, the dosages of omega‐3 FAs administered parenterally or enterally varied from 2.0 to 6.5 g daily, resulting in a decrease in indicators linked to inflammation. However, there were no discernible changes in inflammatory markers when omega‐3 FAs were given to patients before abdominal surgery. The limited number of research studies, which limits how the results can be interpreted (Mohsen et al. 2023), might explain this finding. In patients with acute lung injury (ALI) and acute respiratory distress syndrome (ARDS), immunomodulatory supplementation of omega‐3 and omega‐6 FAs, along with antioxidants delivered through enteral nutrition (EN), did not significantly alter ICU length of stay (LOS), organ failure, or hospital stay when compared to patients given the standard formula, per another systematic review and meta‐analysis (Dushianthan et al. 2019; Li et al. 2015). The omega‐3 FA content of currently marketed enteral formulae in South Korea ranges from 0.8 to 1.7 g/L, while it is still difficult to precisely define the therapeutic impact, and no particular suggested dosage has been identified. About 3 g of fish oil per 100 mL, or 15% of the total volume, is included in commercially available 20% lipid emulsion solutions. Although the manufacturer‐specific composition of FAs in lipid‐based PN formulations varies, preparations based on fish oil usually make up about 15%–20% of the overall lipid content. The Dietary Guidelines for Americans 2015–2020 state that 450–500 mg of omega‐3 FAs per day are needed to sustain optimal physiological performance (Vannice and Rasmussen 2014). The Dietary Reference Intakes for Koreans 2020 state that individuals between the ages of 50 and 64 should consume 500 mg of omega‐3 FAs per day for males and 240 mg per day for women (Hwang et al. 2022).

#### 6.2.2 Nucleotides

Animal studies have shown that long‐term feeding of a nucleotide‐free diet reduces the antibody response to T cell‐dependent antigens in rats, despite the paucity of research specifically concentrating on nucleotide supplementation in people. Immune function quickly improves when nucleotides in the diet are restored (Adjei et al. 1993; Ogoshi et al. 1988). Nucleotide supplementation has been shown in animal experiments to enhance the height of the jejunal villus. Nucleotide‐enriched foods (milk based infant formulas) have been shown to increase intestinal blood flow in human baby models, which may help maintain the integrity of the gut barrier (Carver et al. 2002; Evans et al. 2005; Holen and Jonsson 2004). Nonetheless, the majority of research has paired nucleotides with other immunonutrients like arginine and omega‐3 FAs, which have shown favorable results, including lower infection rates. As a result, it is challenging to attribute these outcomes exclusively to nucleotide effects (Suchner et al. 2000). Although the precise amount of nucleotides in each food item has not been determined, it is thought that healthy people receive 1–2 g daily through their diet. The amount of nucleotides in certain commercially available enteral formulations from foreign sources ranges from 1.2 to 2.8 g/L (Suchner et al. 2000). Nevertheless, it is still unknown what nucleotides are present in local Korean goods.

#### 6.2.3 Glutamine

Glutamate supplementation may have beneficial effects by enhancing immune function and reducing inflammatory responses, according to small‐scale research conducted in critically sick patients in the past (Novak et al. 2002). Over the past 10 years, there has been a significant rise in research in this area. Multicenter trials have shown the adverse effects of glutamine supplementation, despite some single‐center research reporting favorable results (Bistrian 2013). Parenteral nutrition (PN) was used to provide 20.2 g/day of glutamine in one of the largest trials, the Scottish Intensive Care Glutamine or Selenium Evaluative Trial, a double‐blind RCT with 502 critically sick patients. According to the study, glutamine had no discernible impact on the incidence or death rate of infections (Andrews et al. 2011). Similarly, glutamine supplementation was linked to higher mortality without other clinical benefits in the Reducing Deaths due to Oxidative Stress trial (REDOX), a randomized, blinded study that included 1223 patients with multi‐organ failure across 40 intensive care units in Canada, the US, and Europe. It is noteworthy that the REDOX trial's patient cohort included severely sick patients with multiorgan failure, many of whom were enterally fed less than 50% of their nutritional needs. This restriction has an impact on how the results were interpreted (Heyland et al. 2013). Despite being a large‐scale trial, conclusive findings were hindered by the state of the patient population and insufficient nutritional assistance. As a result, more research and meta‐analyses have been carried out. Despite the paucity of available data, glutamine supplementation has demonstrated modest advantages in lowering hospital LOS and infection rates, but not death. Although the ideal dosage and length of treatment have not yet been determined, PN seems to produce better results (Apostolopoulou et al. 2020). According to a meta‐analysis of research, high‐risk patients undergoing elective surgery who were given formulas containing different immunonutrients, such as fish oil and arginine, during the preoperative and postoperative phases experienced a significant decrease in infection rates and LOS, but not in mortality (Drover et al. 2011). Using immunomodulatory formulas throughout the perioperative and postoperative phases resulted in fewer infectious problems, shorter hospital LOS, and fewer overall complications, according to another meta‐analysis of patients having elective gastrointestinal surgery (Osland et al. 2014). These results point to possible advantages for lowering general morbidity. According to the most recent ESPEN guidelines, trauma patients should receive 0.2–0.3 g/kg/day of glutamine by EN during the first 5 days after starting EN. Glutamate should be given for more than 10–15 days in situations of complex wound healing. For patients with burns that encompass more than 20% of their body surface area, 0.3–0.5 g/kg/day is the suggested dosage for 10–15 days. Supplementing with glutamine is not advised for individuals other than those who have burns or injuries. Furthermore, glutamine by PN is not recommended for critically sick patients with complicated diseases, especially those with hepatic or renal failure (Singer et al. 2019).

#### 6.2.4 Arginine

For healthy people, 5–30 g of arginine per day is the suggested dosage. Arginine is usually present in commercially available enteral formulations intended to strengthen the immune system in critically sick patients at concentrations between 0 and 18.7 g/L. Domestic enteral formulae, on the other hand, have much lower arginine amounts, ranging from 0 to 5 g/L. According to international clinical research, critically ill patients who use enteral feeding to reach their target nutritional intake often get 10–30 g of arginine per day (Rosenthal et al. 2016). Because of metabolic instability and inflammatory conditions, it might be difficult to determine the precise dosage for patients who are perioperative or critically unwell. However, because greater dosages may raise the risk of negative effects, arginine supplementation should be within the 10–30 g/day range (McClave et al. 2016; Patel et al. 2016).

#### 6.2.5 Vitamin C and E

According to the 2020 Korean Dietary Reference Intake recommendation, one should consume 100 mg of water‐soluble vitamin C daily, whereas 12 mg of fat‐soluble vitamin E should be consumed daily (Hwang et al. 2022). The vitamin C and vitamin E contents of commercial EN products in Korea vary from 140 to 500 mg per 1000 kcal and 10 to 50 mg α‐TE per 1000 kcal, respectively (Jayasekara et al. 2020; Kim et al. 2022).

## 7 Challenges, Limitations, and Future Directions

- Deficiencies of essential nutrients like zinc and vitamin C are reported by studies among diabetic patients (e.g., foot ulcers) or chronic wounds.
- Lack of good, detailed data hinders standardized regimes of micronutrient supplementation throughout wound healing.
- Without such a comprehensive profile, physicians cannot properly tailor nutritional therapy, which could affect wound healing outcomes.
- There are considerable variations in the approaches employed to measure nutritional immunity, which result in a variety of outcomes from various studies.
- While some suggested instruments have displayed potential, including the Zinc‐to‐CRP ratio, serum ferritin adjusted for inflammation, and mixed evaluation methods like the Subjective Global Assessment (SGA) or Prognostic Inflammatory and Dietary Index, none are yet established for regular application in wound therapy or infectious illness settings.
- The critical necessity of standardized recommendations to increase uniformity and practical application is highlighted by the lack of verified, wound‐specific nutritional immunity panels.
- Heterogeneity makes comparing research more difficult, and studies provide varying suggestions for best practice.
- Determination of nutritional status in wound healing would become more clinically relevant and reliable with standardized testing.
- With increased standardization, immune reactions of chronic or infected wound patients might be handled more easily, and treatments are tailored.
- Cross‐sectional study designs, which provide an overview of the nutritional status and wound state at one particular point, are frequently used in the majority of the research currently available on dietary therapies and wound healing.
- Although these studies can find correlations, they are unable to document changes over time or prove a link between supplementary micronutrients and recuperation results. It is hard to say whether dietary deficits are a result of long‐term wounds or a contributing factor for inadequate healing without longitudinal follow‐up or controlled therapies.
- The necessity for prospective cohort research and randomized controlled trials to fully clarify the linear and molecular connections between wound recovery and nutrition is made apparent by this methodological restriction.
- Studies that examine the biological processes involved in wound healing and the role of specific nutrients could provide valuable information.
- With longitudinal and mechanistic studies, personalized dietary therapies will be provided depending on the individual patient's nutritional requirements and healing pattern.
- Multi‐omics, that is, metallomics and ionomics, could ascertain the complex biological wound healing process.
- These techniques allow for the collection of more detailed information regarding gene regulation, metal ions, and wound healing mechanisms.
- Integrating data from numerous omics platforms may prove to be challenging, and the heterogeneity and complexity of the data present analytical challenges.
- Established data processing measures, in addition to sophisticated instruments like machine learning and holistic statistical frameworks like multi‐block or Bayesian techniques, can be used to overcome this obstacle.
- The interpretation of intricate biological information can be facilitated by systems such as DIABLO and MOFA (Multi‐Omics Factor Analysis), which are especially useful in spotting major trends across various omics layers.
- New biomarkers and drug targets for healing wounds are available through multi‐omics technology.
- AI can blend complex data from immune response, diet, and microbiome profiles to attempt to predict how a wound will heal.
- By utilizing combined data, AI models can help doctors make more accurate, personalized treatment decisions.
- AI will study the link between diet, immunological reactions, and diversity of microbiomes to create stronger treatment regimens.

## 8 Conclusion

Wound infections are dynamic communities in which host defense mechanisms and microbial tactics are in constant interaction, and nutritional immunity is central to this conflict. By sequestering essential trace elements like iron, zinc, copper, and manganese, the host generates an inhospitable environment for the pathogen, restricting its metabolic activity and cell division. But the arms race of evolution has armed the pathogen with successful mechanisms to evade them, including secretion of biofilm and high‐affinity nutrient‐binding proteins. Concurrently, the idea of immunonutrition stands as a preventative measure to promote host recovery and immunity through directed dietary therapies. Nutrients such as omega‐3 fatty acids, glutamine, arginine, nucleotides, and vitamins C and E not only help in wound healing but also in immune function, preventing susceptibility to infection and aiding in the regeneration of tissues.

## Author Contributions

**Chaoming Chen:** writing – original draft (equal), writing – review and editing (equal). **Xuanfan Hu:** validation (equal). **Da He:** conceptualization (equal), methodology (equal). **Xuemei He:** software (equal), validation (equal). **Lan Shen:** supervision (equal).

## Ethics Statement

The authors have nothing to report.

## Consent

All authors are willing for publication of this manuscript.

## Conflicts of Interest

The authors declare no conflicts of interest.

## Data Availability

Even though adequate data has been given in the form of tables and figures, all authors declare that if more data is required, then the data will be provided on a request basis.
